# Point-of-Care Ultrasound: A High-Tech Solution for Low- and Middle-Income Countries

**DOI:** 10.7759/cureus.83520

**Published:** 2025-05-05

**Authors:** Alexander S Doyal, Philip Sholes, Elizabeth Drum, Birhane Tesfay, Bantayehu Sileshi

**Affiliations:** 1 Anesthesiology, University of North Carolina at Chapel Hill School of Medicine, Chapel Hill, USA; 2 Anesthesiology, Baptist Hospital, Pensacola, USA; 3 Anesthesiology, Children's Hospital of Philadelphia, Philadelphia, USA; 4 Anesthesiology, Addis Ababa Medical University College, Addis Ababa, ETH; 5 Anesthesiology, Vanderbilt University Medical Center, Nashville, USA

**Keywords:** anesthesiology, healthcare delivery, healthcare disparities, low- and middle-income countries (lmics), medical imaging, point-of-care ultrasound (pocus), procedural guidance, real-time imaging, training initiatives

## Abstract

Point-of-care ultrasound (POCUS) is a transformative tool for anesthesiology in low- and middle-income countries (LMICs), where limited access to advanced medical imaging, unreliable power, and scarce resources hinder patient care. This technology's portability, affordability, and diagnostic versatility address critical gaps, enabling better preoperative assessment, intraoperative monitoring, and emergency interventions. POCUS facilitates procedures such as catheter placement and nerve blocks, diagnoses conditions such as pneumothorax, and evaluates cardiac function, all without ionizing radiation. Training initiatives, including hands-on workshops and tele-mentoring, empower providers in resource-limited settings. By integrating POCUS into anesthesiology practices, LMICs can enhance patient outcomes and elevate healthcare delivery.

## Editorial

Introduction

Low- and middle-income countries (LMICs) are often heavily challenged by their lack of medical resources and access to medical imaging modalities. Our own specialty of anesthesiology is especially affected by these limitations. Point-of-care ultrasound (POCUS) has emerged as a versatile and transformative tool in anesthesiology, offering potential benefits for resource-limited settings. In this article, we explore the value of POCUS in anesthesiology for LMICs. We will delineate the challenges faced in LMICs and explore the benefits of POCUS in these settings, highlighting the portability, cost-effectiveness, diagnostic capabilities, procedural usefulness, and ease of learning, all towards the ultimate goal of improving patient outcomes [1,2].

Challenges in anesthesiology in LMICs

Before delving into the context-specific utility of POCUS, it is essential to understand the unique challenges faced by anesthesiologists and healthcare workers in LMICs. Many facilities in these regions lack access to advanced imaging technologies such as CT scans and MRI machines, making preoperative assessment and intraoperative monitoring more challenging. Furthermore, many LMICs have an unreliable power grid, which may lead to prolonged and widespread electricity outages. There is often limited access to the internet via wifi or cellular networks. Many providers lack personal laptops or smartphones. Regular maintenance and repair of medical equipment may be lacking as there are very limited skilled technical workers and an interrupted supply chain for parts. On a recent trip to the capital city of an LMIC South African nation, we witnessed that the CT scanner was inoperable for the last three months, without MRI available in the entire nation, and their sole portable chest X-ray machine was broken. This necessitated a long patient transport from the ICU for any radiological study. These limitations of resources make the care of critically ill patients very challenging, and practitioners rely heavily on physical examination findings.

Advanced anesthesia equipment and monitoring devices (e.g., cardiac output) are expensive to purchase and maintain in good working order. Thus, their availability may be limited to a single operating room (OR) or they may not be available at all. The more basic technology that we often rely on, such as pulse oximetry, capnography, oxygen sensors, and volatile gas analysis, may also be unavailable. This void of technology causes skilled healthcare providers to rely heavily on physical examination and clinical observation of the patient [3].

Educational opportunities may be limited in LMICs. Anesthesiologists and anesthesiology healthcare officers in these regions frequently have limited opportunities for continuing medical education and training, reducing their exposure to advanced techniques and technologies.

Advantages of POCUS in LMICs

In anesthesiology, POCUS is becoming more widespread globally, and this technology is particularly valuable in low-resource settings due to several key advantages it offers. POCUS machines are durable, cost-effective, compact, lightweight, and portable, making them highly desirable in resource-limited settings. The commonly used Butterfly iQ+, GE Vscan Air, and Phillips Lumify ultrasound machines can plug into a smartphone or tablet, obviating the need for a large wheeled machine [4]. When compared to larger ultrasound machines or other imaging modalities such as CT and MRI, POCUS devices are relatively affordable, making them much more accessible. A typical handheld POCUS device costs approximately $2,000-5,000 USD. This is not an insignificant cost to many LMICs, but significantly less than the $0.5-3 million USD for a CT or MRI machine.

POCUS can aid anesthesiologists in many procedures, such as central venous catheterization, arterial line placement, peripheral intravenous line placement, peripheral nerve blocks, and neuraxial anesthesia, improving the success rate and reducing the risk of complications [5-8].

POCUS can aid in the diagnosis of many critical conditions. This real-time imaging and diagnosis allows for time-sensitive treatment and provides timely interventions to potentially improve patient outcomes. POCUS can quickly identify pneumothorax and pleural effusion, both of which can have significant implications for anesthetic management. This, for example, was useful in the OR and ICU setting for the prompt diagnosis of a hemopneumothorax in a polytrauma patient. POCUS is also a useful tool for placing chest tubes, as well as confirming their proper placement and adequate drainage [9]. Unlike X-rays or CT scans, POCUS is non-invasive and does not involve exposure to ionizing radiation, making it safer for both patients and healthcare providers.

POCUS can help evaluate cardiac function, identifying conditions such as pericardial effusion or cardiac tamponade that require prompt intervention. Cardiac POCUS can also assist in differentiating between severe hypovolemia and cardiogenic shock in the perioperative trauma setting [10]. Learning how to perform a FAST examination can also aid clinicians in detecting an occult abdominal bleed in trauma [11]. In addition to identifying lung pathologies, pulmonary POCUS can be used to confirm proper endotracheal tube placement [12].

**Figure 1:**
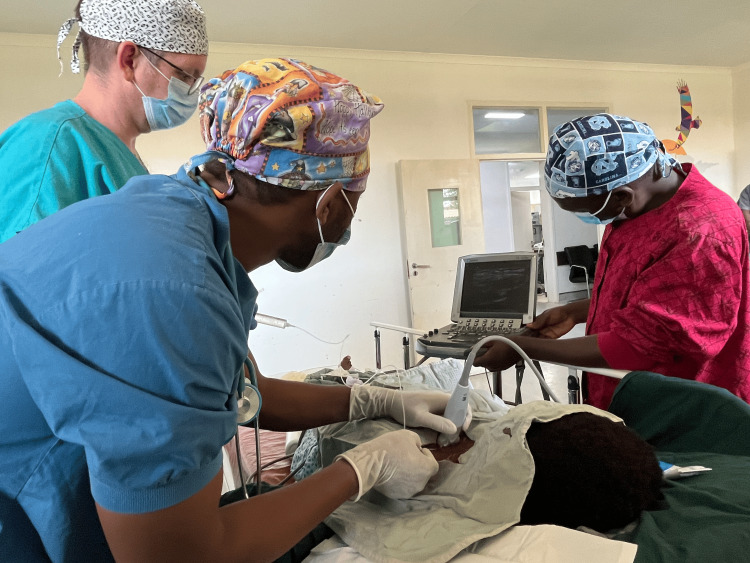
Hands-on education of POCUS being utilized to place a peripheral nerve block Consent was obtained from all healthcare workers shown POCUS, point-of-care ultrasound

Taken together, POCUS can serve as a valuable tool for anesthesia providers in the OR, especially when advanced equipment or portable X-ray is unavailable (Figure 1).

Existing educational barriers are somewhat minimal for POCUS as it is easy to learn, and learners can quickly gain proficiency. There are many Free Open Access Medical Education (FOAM) resources available for LMICs. This includes several online training modules for POCUS. However, such tools often require access to the internet with sufficient bandwidth to stream videos, thus limiting their access. Several training initiatives are currently underway in the developing world to increase both access to the technology and how to use it to its maximum benefit. During one recent training session, the author was impressed by how quickly the healthcare providers in the OR and ICU were able to learn and implement POCUS into their practice.

As a relevant clinical example, during a recent educational trip, after a didactic session on how to diagnose lung pathology with POCUS, the healthcare officers, who also serve as ICU attendings, were able to quickly utilize the new technology. A trauma patient who had bilateral pneumothoraces and pleural effusions was in need of a chest tube based on chest X-ray. Using POCUS, the providers were able to diagnose the condition, use ultrasound to select a site for chest tube insertion (instead of blind placement), confirm appropriate chest tube placement, and serially assess the size of each effusion and pneumothorax. This example highlights the clinical utility as well as the ease of implementation of this new technology in the current medical practice.

**Figure 2:**
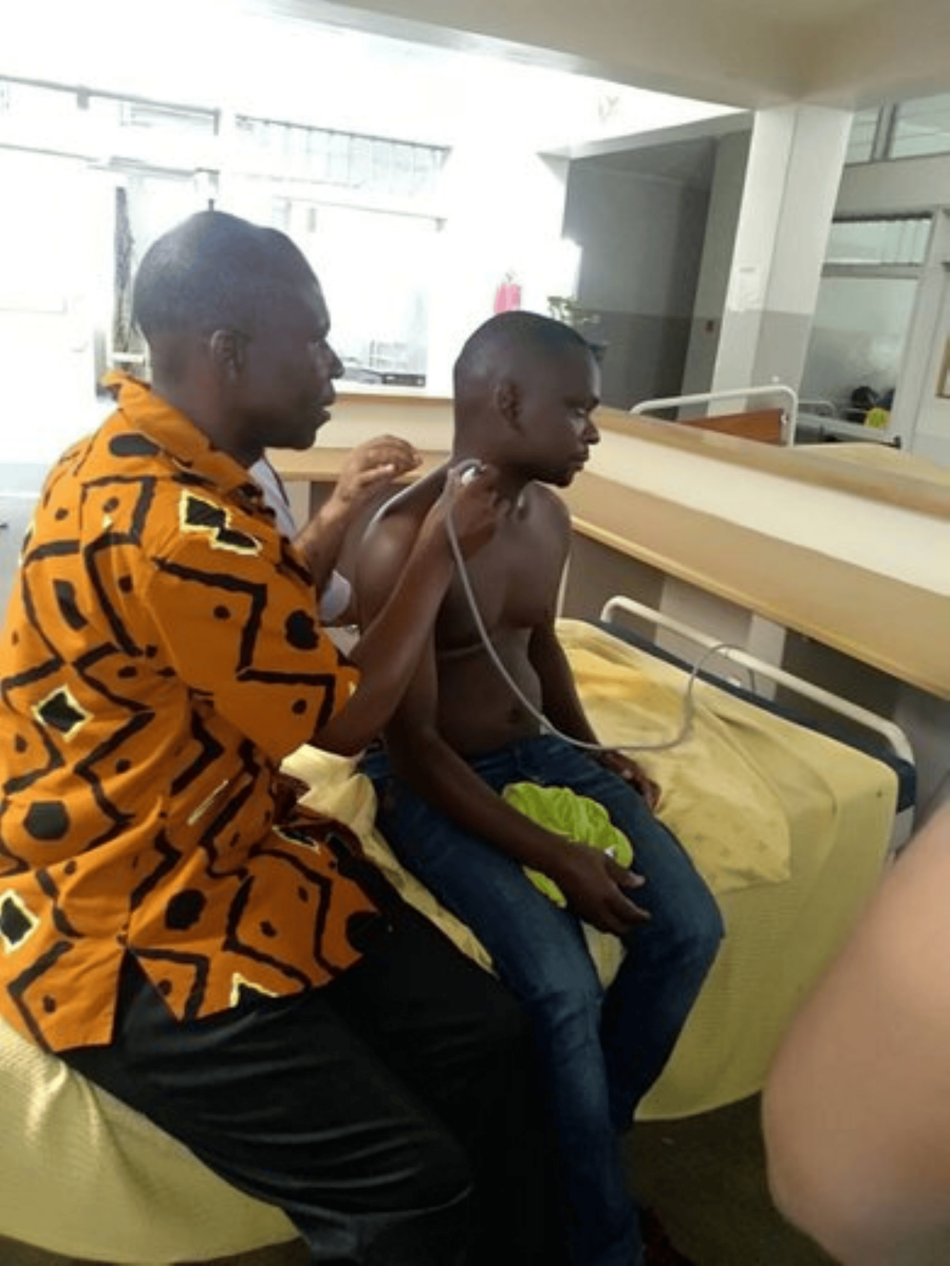
Two anesthesia healthcare officers practicing obtaining POCUS views on one another Consent was obtained from all healthcare workers shown POCUS, point-of-care ultrasound

Hands-on training sessions are pivotal, emphasizing the use of portable ultrasound devices that are accessible and user-friendly. Simulation-based learning, often using low-cost or improvised models, allows learners to practice image acquisition and interpretation in a controlled environment. Peer-to-peer teaching and mentorship play a critical role, leveraging the expertise of local healthcare providers who can train others within their community. Additionally, remote learning tools, such as online courses and tele-mentoring, are increasingly utilized to provide continuous education and support, ensuring that practitioners in LMICs can confidently integrate POCUS into their clinical practice (Figure 2).

To harness the full potential of POCUS in anesthesiology, appropriate training and skill development are essential for healthcare providers. Future educational efforts should include POCUS training in the curriculum of medical schools and anesthesiology residency programs in LMICs. Online educational resources (often built into the device programming) and international telemedicine platforms can facilitate remote training and consultation with experienced POCUS practitioners from around the world [13]. Robust platforms that allow educational training, remotely controlled image optimization, and viewing of bedside ultrasound images have been developed by companies such as Butterfly. This could be useful in collaboration between healthcare entities in developed nations and LMICs to facilitate training, quality control of image capturing, and diagnostic interpretation.

Conclusion

It is clear that POCUS has emerged as a valuable and versatile tool in anesthesiology. Its portability, cost-effectiveness, real-time imaging capabilities, and diagnostic applications make it a crucial asset for anesthesiologists striving to provide optimal care under challenging circumstances found in LMICs. By addressing the unique challenges and promoting training initiatives, the integration of POCUS into anesthesiology practice may significantly improve patient outcomes and enhance healthcare delivery in regions with limited resources.
